# Assessing potentiation of the (α4)3(β2)2 nicotinic acetylcholine receptor by the allosteric agonist CMPI

**DOI:** 10.1016/j.jbc.2021.101455

**Published:** 2021-11-30

**Authors:** Farah Deba, Kemburli Munoz, Eloisa Peredia, Gustav Akk, Ayman K. Hamouda

**Affiliations:** 1 Department of Pharmaceutical Sciences, The University of Texas at Tyler, Tyler, Texas, USA; 2 Department of Pharmaceutical Sciences, Texas A&M HSC, Kingsville, Texas, USA; 3 Department of Anesthesiology, Washington University in St. Louis, St. Louis, Missouri, USA; 4 The Taylor Family Institute for Innovative Psychiatric Research, Washington University in St. Louis, St. Louis, Missouri, USA

**Keywords:** positive allosteric modulators, nicotinic acetylcholine receptors, CMPI, MWC model, ABS, agonist binding site, ACh, acetylcholine, CMPI, 3-(2-chlorophenyl)-5-(5-methyl-1-(piperidin-4-yl)-1H-pyrrazol-4-yl)isoxazole, dFBr, desformylflustrabromine, MWC, Monod-Wyman-Changeux, nAChR, nicotinic acetylcholine receptor, PAMs, positive allosteric modulators

## Abstract

The extracellular domain of the nicotinic acetylcholine receptor isoforms formed by three α4 and two β2 subunits ((α4)3(β2)2 nAChR) harbors two high-affinity “canonical” acetylcholine (ACh)-binding sites located in the two α4:β2 intersubunit interfaces and a low-affinity “noncanonical” ACh-binding site located in the α4:α4 intersubunit interface. In this study, we used ACh, cytisine, and nicotine (which bind at both the α4:α4 and α4:β2 interfaces), TC-2559 (which binds at the α4:β2 but not at the α4:α4 interface), and 3-(2-chlorophenyl)-5-(5-methyl-1-(piperidin-4-yl)-1H-pyrrazol-4-yl)isoxazole (CMPI, which binds at the α4:α4 but not at the α4:β2 interface), to investigate the binding and gating properties of CMPI at the α4:α4 interface. We recorded whole-cell currents from *Xenopus laevis* oocytes expressing (α4)3(β2)2 nAChR in response to applications of these ligands, alone or in combination. The electrophysiological data were analyzed in the framework of a modified Monod–Wyman–Changeux allosteric activation model. We show that CMPI is a high-affinity, high-efficacy agonist at the α4:α4 binding site and that its weak direct activating effect is accounted for by its inability to productively interact with the α4:β2 sites. The data presented here enhance our understanding of the functional contributions of ligand binding at the α4:α4 subunit interface to (α4)3(β2)2 nAChR-channel gating. These findings support the potential use of α4:α4 specific ligands to increase the efficacy of the neurotransmitter ACh in conditions associated with decline in nAChRs activity in the brain.

Neuronal nicotinic acetylcholine receptors (nAChRs) are pentameric ligand-gated ion channels formed of identical or distinct but homologous subunits (α2–α10 and β2–β4). Homomeric α7 and heteromeric α4β2 nAChRs are the major subtypes in the brain (1, 2, 3). Postsynaptic nAChRs mediate fast synaptic transmission, whereas presynaptic nAChRs modulate the release of many neurotransmitters (4, 5). Thus, nicotinic receptors are involved in complex brain functions, including cognition, pain perception, and neuronal survival during aging (3, 5). Furthermore, nAChRs mediate the behavioral effects of nicotine, the major addictive component of tobacco smoking, and are considered a major molecular target for pharmacotherapeutic interventions to manage nicotine dependence (6, 7). Therapeutics targeting the nAChRs also have potential clinical relevance in reducing chronic pain and slow cognitive decline associated with neuropsychiatric conditions (8).

Neuronal nAChRs consisting of α4 and β2 subunits assemble in two stoichiometries: (α4)2(β2)3 and (α4)3(β2)2 (9). The initial pharmacological distinction between the (α4)2(β2)3 and (α4)3(β2)2 isoforms was based on their sensitivity to acetylcholine (ACh). ACh potency (*EC*_*50*_) is ∼1 μM at the (α4)2(β2)3 nAChR and ∼100 μM at the (α4)3(β2)2 nAChR; the two isoforms are thus referred to as the high- and low-sensitivity α4β2 nAChRs (10). Both isoforms contain two high-affinity agonist binding site (ABS) located in the two α4:β2 intersubunit interfaces in the extracellular domain, whereas the (α4)3(β2)2 nAChR has a third, low-affinity ACh-binding site located in the α4:α4 subunit interface. Subsequent studies on heterologously expressed (α4)3(β2)2 and (α4)2(β2)3 nAChRs revealed a number of key differences in channel functional properties and pharmacological selectivity to exogenous nAChR ligands (11, 12, 13, 14, 15). Assembly of both α4β2 nAChR isoforms has been reported *in vivo* (16). The (α4)3(β2)2 nAChR isoform is considered the major isoform expressed in the cortex (17), whereas the (α4)2(β2)3 nAChR isoform contributes to nicotine dependence and is selectively upregulated and stabilized after chronic nicotine exposure (18, 19, 20, 21).

High-throughput screening has identified several nAChR subtype-selective positive allosteric modulators (PAMs) (12, 22, 23, 24, 25, 26). CMPI (3-(2-chlorophenyl)-5-(5-methyl-1-(piperidin-4-yl)-1H-pyrrazol-4-yl)isoxazole) and NS9283 (3-[3-(3-pyridinyl)-1,2,4-oxadiazol-5-yl]benzonitrile) have been identified as potent nAChR PAMs that preferentially potentiate the (α4)3(β2)2 isoform (12, 23). At first glance, the pharmacology of CMPI and NS9283 (location of binding site and effect on ACh concentration-response curve) at the (α4)3(β2)2 nAChR resembles that of benzodiazepines at the GABA_A_ receptor (27). However, unlike benzodiazepines and GABA at the GABA_A_ receptor, CMPI, NS9283, and ACh share an overlapping binding site at the α4:α4 subunit extracellular interface in the (α4)3(β2)2 nAChR (12, 28), raising the possibility that CMPI and NS9283 could act as agonists at the α4:α4 site. Indeed, NS9283, which binds at the α4:α4 interface and at the β2:α4 pseudo-agonist site (29), was found to enhance nAChR (α4)3(β2)2 channel activity by transitioning the channel into a preactivated state (30).

In this study, we investigated the properties of CMPI and other nAChR ligands to delineate the pharmacology of the α4:α4 binding site and to elucidate allosteric interaction between the α4:β2 and α4:α4 interface-binding sites. Current responses from the (α4)3(β2)2 nAChR elicited by a series of nAChR agonists, alone or in combination with CMPI, were analyzed using a modified Monod-Wyman-Changeux (MWC) allosteric activation model (31, 32, 33). Our results indicate that CMPI is a high-affinity, high-efficacy agonist at the α4:α4 binding site. It binds to the α4:α4 interface with a higher affinity than ACh, cytisine, or nicotine, and efficaciously potentiates receptor responses to subsaturating concentrations of these agonists. The gating efficacy of CMPI at the α4:α4 site is equivalent to that of ACh, whereas weak direct activation of the (α4)3(β2)2 nAChR in the presence of CMPI is accounted for by a single binding site mediating its action. Thus, CMPI enhances channel gating triggered by ACh at the α4:β2 sites by providing ligand occupancy at the α4:α4 site, which is otherwise vacant or only occupied at very high (hundreds of μM) ACh concentrations. The data presented here enhance our understanding of ligand-binding properties and functional contributions of the “noncanonical” α4:α4 subunit interface to nAChR channel gating and facilitate structure-based design of novel therapeutics that selectively target the (α4)3(β2)2 nAChR.

## Results

### CMPI potentiation of (α4)3(β2)2 nAChR currents induced by subsaturating and saturating agonist concentrations

Our initial characterization of CMPI-induced potentiation of the (α4)3(β2)2 nAChR employed the neurotransmitter ACh as the agonist. Coapplication of CMPI increased current responses to *EC*_*10*_ (10 μM) ACh to 386 ± 15% of control with a potentiation *EC*_*50*_ of 0.18 ± 0.03 μM (28). CMPI at 1 μM produced a ∼100 fold left-shift of the ACh concentration-response curve enhancing ACh potency with no apparent effect on the ACh maximal response (15, 28). ACh is known to bind with high affinity at the α4:β2 agonist-binding site (ABS) and with a lower affinity at the α4:α4 ABS (34).

**Figure 1:**
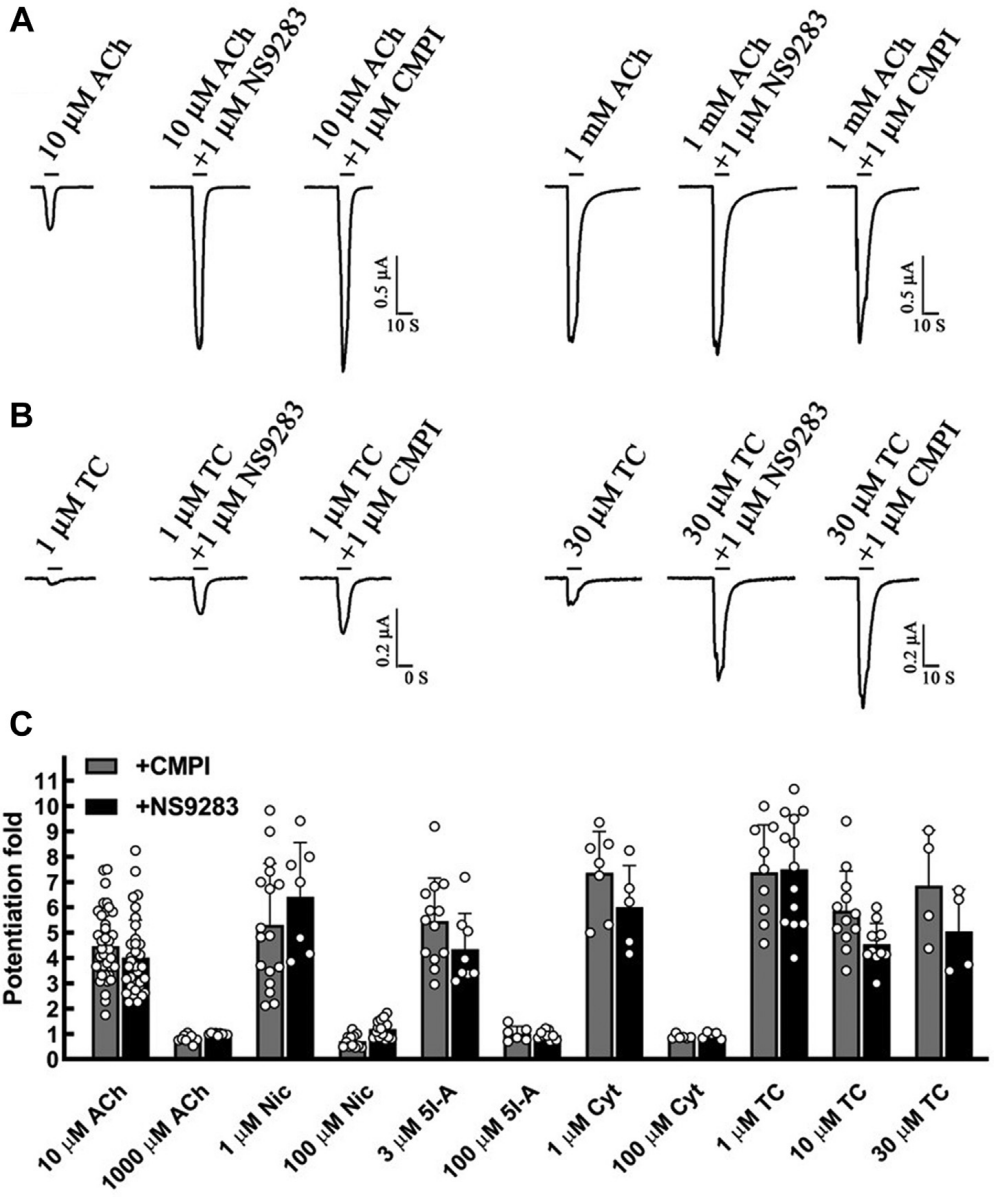
**Effects of CMPI and NS9283 on (α4)3(β2)2 nAChR current responses elicited by subsaturating and saturating agonist concentrations.** The traces show whole-cell currents elicited by 10 s applications of low or saturating concentrations of various nAChR agonists alone or in the presence of 1 μM of CMPI or NS9283. Representative traces for ACh and TC-2559 are shown in (*A* and *B*), respectively. *C*, for each agonist concentration, the peak currents were normalized to the peak current elicited by agonist alone. The data obtained from several oocytes were plotted as mean ± SD with values of individual oocytes are shown in *open circles*. The probability (*P*) that calculated potentiation ratio differs from no potentiation (PR = 1) was analyzed using one-way ANOVA with multiple comparisons *versus* control group (Holm–Sidak method, SigmaPlot, and Systat Software Inc). The effects of 1 μM of CMPI or NS9283 on current elicited by 10 μM ACh, 1 μM Nicotine, 3 μM 5I-A85380, 1 μM Cytisine, 1 μM TC 2559, 10 μM TC 2559, and 30 μM TC 2559 were statistically significant with a *p* < 0.001. The data for 10 μM ACh + 1 μM CMPI and 10 μM ACh + 1 μM CMPI contain data from oocytes that were reported previously (28). ACh, acetylcholine; CMPI, 3-(2-chlorophenyl)-5-(5-methyl-1-(piperidin-4-yl)-1H-pyrrazol-4-yl)isoxazole; nAChR, nicotinic acetylcholine receptor.

To expand these studies to other nAChR agonists and to mechanistically characterize the interaction between drugs that bind at the α4:α4 ABS and agonists that interact with the α4:β2 sites, we first determined the effect of 1 μM (a saturating concentration) CMPI on (α4)3(β2)2 nAChR currents induced by subsaturating or saturating concentrations of a series of nAChR agonists (Fig. 1). In parallel, we determined the effect of NS9283, another nAChR PAM, that binds at the α4:α4 subunit extracellular interface and thus preferentially potentiates the (α4)3(β2)2 isoform. CMPI and NS9283 potentiated responses to ACh, nicotine, cytisine, and TC-2559 when the agonists were applied at subsaturating concentrations (potentiation folds in the presence of 1 μM CMPI or NS9283 were statistically significantly different from no potentiation with a *p* < 0.001). In contrast, CMPI and NS9283 did not potentiate responses to saturating concentrations of the tested agonists, except for TC-2559. The effects of 1 μM CMPI or NS9283 on currents elicited by 10 μM TC-2559 and 30 μM TC-2559 were statistically significant with a *p* < 0.001.

**Figure 2:**
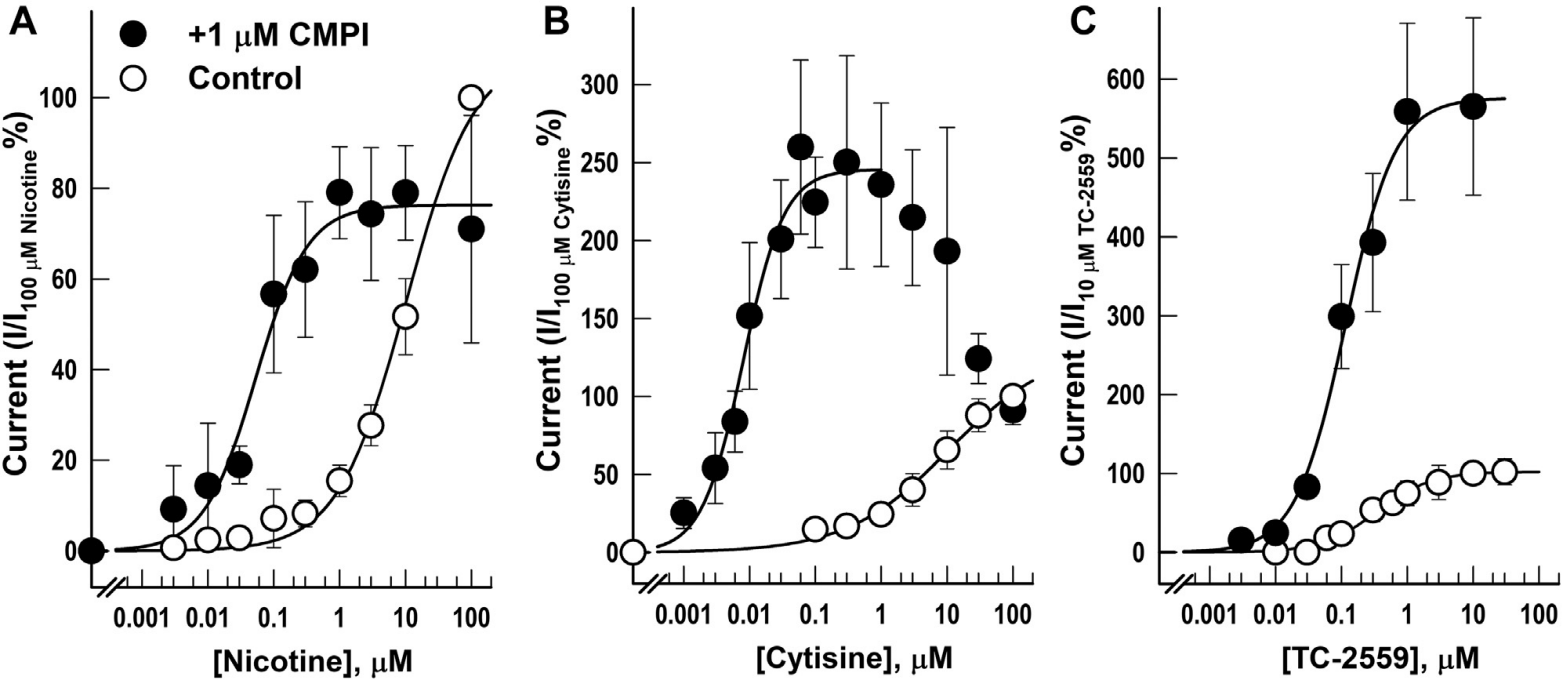
**Effects of coapplication of CMPI on the concentration-response curves of nicotine, cytisine, and TC-2559 at the (α4)3(β2)2 nAChR.** The whole-cell current elicited by 10 s applications of increasing concentrations of nicotine (*A*), cytisine (*B*), or TC-2559 (*C*) in the absence or presence of 1 μM CMPI. For each drug application, the peak currents were normalized to the peak current elicited by 100 μM nicotine (*A*), 100 μM cytisine (*B*), or 10 μM TC-2559 (*C*) applied in the same recording run. The recording runs from same oocyte were combined and each point plotted are mean ± SD of data obtained from at least three oocytes. The data were fit to a single site model using Equation 1. CMPI, 3-(2-chlorophenyl)-5-(5-methyl-1-(piperidin-4-yl)-1H-pyrrazol-4-yl)isoxazole; nAChR, nicotinic acetylcholine receptor.

The effects of CMPI on the concentration-response curves for nicotine, cytisine, and TC-2559 are shown in Figure 2. The coapplication of 1 μM CMPI produced a left-shift in the nicotine concentration-response curve (Fig. 2*A*), enhancing the potency of nicotine (*EC*_*50*_ decreased from 10 ± 3 μM to 0.05 ± 0.01 μM). At saturating nicotine concentrations, the effect of CMPI was reduced, and the observed maximal responses (*E*_*max*_ + 1 μM CMPI) was 76 ± 3% of control (*i.e.*, nicotine alone). Cytisine activated (α4)3(β2)2 nAChRs with an *EC*_*50*_ of 11 ± 6 μM (Fig. 2*B*). In the presence of CMPI, the cytisine concentration-response curve was biphasic. At cytisine concentrations below 1 μM, CMPI enhanced cytisine response by increasing both its potency and efficacy (*EC*_*50*_ + 1 μM CMPI = 0.008 ± 0.001 μM; *E*_*max*_ + 1 μM CMPI = 246 ± 10%). However, at cytisine concentrations greater than 1 μM, CMPI potentiation of cytisine responses gradually declined reaching no effect at 100 μM cytisine (*I*_*100 μM cytisine*_ + 1 μM CMPI = 91 ± 5%). In contrast, CMPI significantly increased TC-2559 efficacy at (α4)3(β2)2 nAChRs (*E*_*max*_ + 1 μM CMPI = 576 ± 26% of that of 100 μM TC-2559 control) with less pronounced effects on TC-2559 potency (*EC*_*50*_ = 0.34 ± 0.04; *EC*_*50*_ + 1 μM CMPI = 0.12 ± 0.02 μM) (Fig. 2*C*). In parallel experiments, NS9283 produced similar effects on the concentration-response curves of nicotine (*EC*_*50*_ + 1 μM NS9283 = 0.43 ± 0.04 μM; *E*_*max*_ + 1 μM NS9283 = 141 ± 9%), cytisine (*EC*_*50*_ + 1 μM NS9283 = 0.013 ± 0.002 μM; *E*_*max*_ + 1 μM NS9283 = 188 ± 5%; *I*_*100 μM cytisine*_ + 1 μM NS9283 = 113 ± 9%), and TC-2559 (*EC*_*50*_ + 1 μM NS9283 = 0.1 ± 0.02 μM; *E*_*max*_ + 1 μM NS9283 = 468 ± 30%) (data not shown).

**Figure 3:**
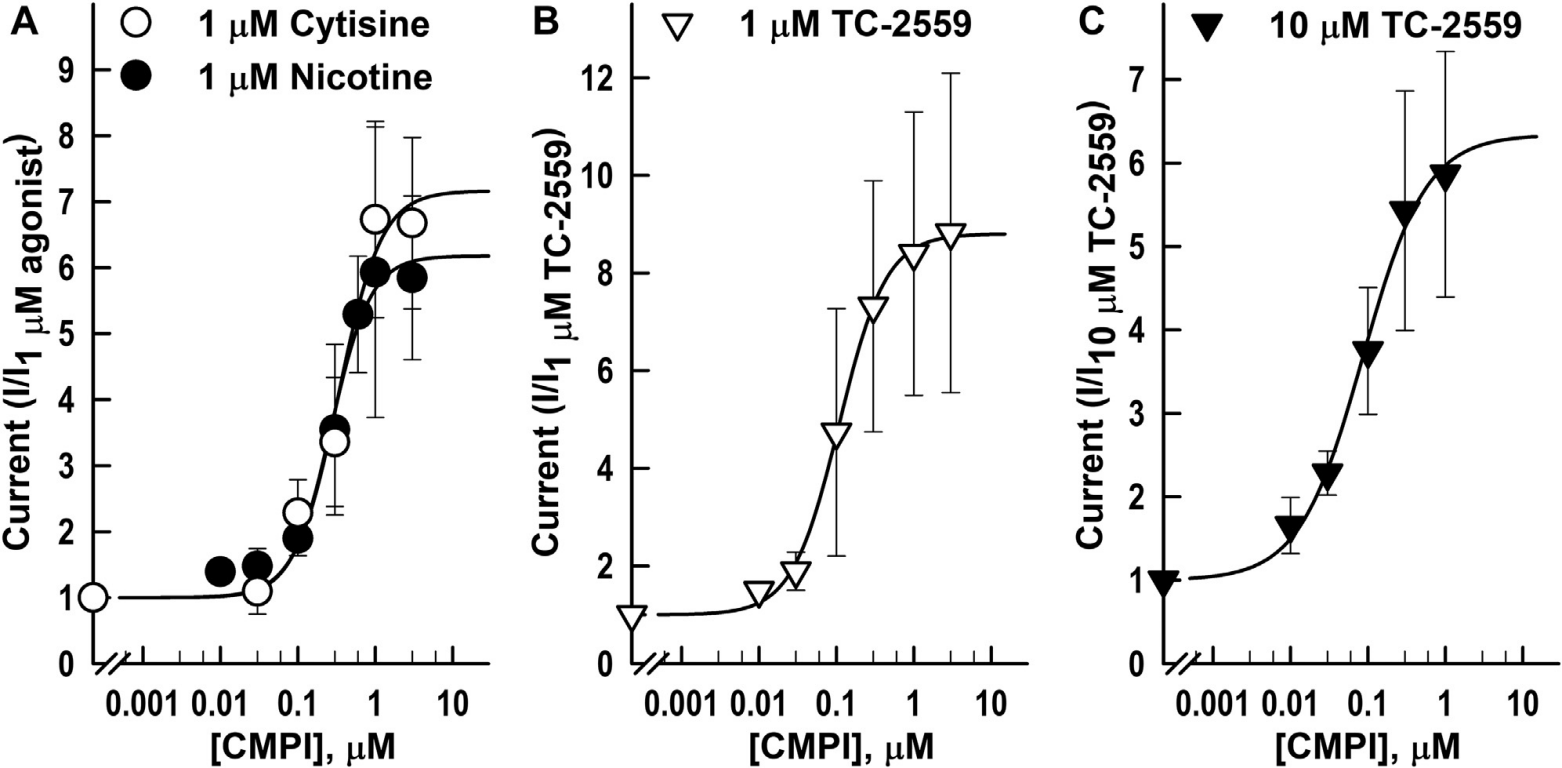
**Effect of coapplication of CMPI with agonist on (α4)3(β2)2 nAChR.** CMPI concentration-dependent potentiation of (α4)3(β2)2 nAChR current responses induced by 1 μM cytisine or nicotine (*A*), 1 μM TC-2559 (*B*), or 10 μM TC-2559 (*C*) in the absence and presence of increasing concentrations of CMPI. The peak currents were normalized to the peak current elicited by agonist alone applied in the same recording run. Recording runs from the same oocyte were combined and each point plotted are mean ± SD of data obtained from at least three oocytes. The data were fit to a single site model using Equation 1. CMPI, 3-(2-chlorophenyl)-5-(5-methyl-1-(piperidin-4-yl)-1H-pyrrazol-4-yl)isoxazole; nAChR, nicotinic acetylcholine receptor.

Dissimilar effects of CMPI on the concentration-response curves for nicotine, cytisine, and TC-2559 were not solely reflections of the differences in agonist efficacies. There was no correlation between agonist efficacy and the extent of CMPI potentiation (*E*_*max*_) or CMPI concentration that produced half-maximum potentiation (*EC*_*50*_) (Fig. 3). The current responses produced by a saturating concentration (100 μM) of cytisine and nicotine were 10 ± 1 and 73 ± 3% of current elicited by 1 mM ACh. CMPI potentiated the responses induced by subsaturating concentrations of cytisine and nicotine (which elicited 2.3 ± 0.4 and 2.9 ± 0.4% of the current response to 1 mM ACh, respectively) with similar potencies and efficacies. CMPI EC_50_s for potentiation of receptors activated by cytisine and nicotine were 0.34 ± 0.08 μM and 0.27 ± 0.04 μM and maximal potentiation was 716 ± 59 and 618 ± 34% of control, respectively (Fig. 3*A*). On the other hand, CMPI potentiated (α4)3(β2)2 nAChR current responses to subsaturating and saturating concentrations of TC-2559 (Fig. 3, *B* and *C*) with potentiation EC_50_s of 0.11 ± 0.01 and 0.09 ± 0.04 μM and maximal potentiation of 880 ± 13 and 634 ± 94% (equivalent to 44.6 and 48.4% of current elicited by 1 mM ACh), respectively. NS9283 potentiated (α4)3(β2)2 nAChR responses induced by 1 μM or 10 μM TC-2559 with potentiation *EC*_*50*_s of 1.9 ± 0.2 and 1.8 ± 0.12 μM and maximal potentiation of 1800 ± 307 and 1522 ± 41%, respectively (data not shown).

### CMPI potentiation of TC 2559-induced currents in the (α4H116A)3(β2)2 nAChR

**Figure 4:**
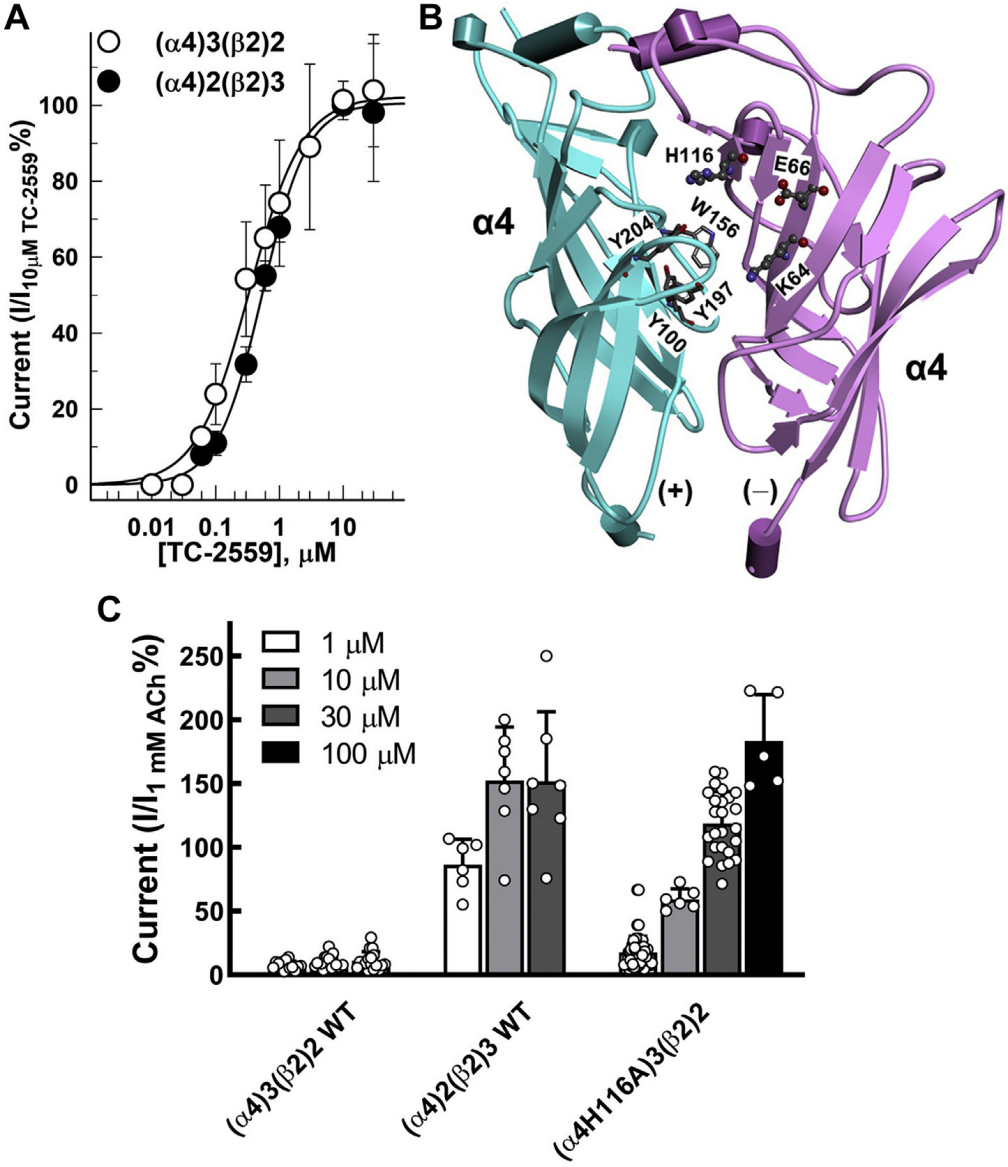
**TC-2559 efficacy at (α4)2(β2)3, (α4)3(β2)2, and (α4H116A)3(β2)2 nAChRs.***A*, TC-2559 concentration-response curves in (α4)2(β2)3 and (α4)3(β2)2 nAChRs. *B*, a side view of the (α4)3(β2)2 nAChR (PDB accession number 6CNK) (46) showing the extracellular domain of the two adjacent α4 subunits forming the α4:α4 interface. The amino acid residues Lysine 64 (K64), Glutamate 66 (E66), and Histidine 116 (H116) that contribute to the α4(−) face of the α4:α4 interface are shown in *stick* and *ball* format. Also shown in *stick* format aromatic amino acid residues that form the (+) face of the agonist-binding site. *C*, bar graph showing the peak currents elicited in response to 1, 10, 30, or 100 μM TC-2559 normalized to the peak currents elicited by 1 mM ACh applied in the same recording run. Shown are the mean ± SD of N oocytes for each TC-259 concentration. The number of oocytes were for (α4)2(β2)3 nAChR (3/3/7) and for (α4)3(β2)2 nAChR (13/20/17) for 1, 10, or 30 μM TC-2559, respectively. The number of oocytes were for (α4H116A)3(β2)2 nAChR (50/6/26/6) for 1, 10, 30, or 100 μM TC-2559, respectively. ACh, acetylcholine; CMPI, 3-(2-chlorophenyl)-5-(5-methyl-1-(piperidin-4-yl)-1H-pyrrazol-4-yl)isoxazole.

TC-2559 activates (α4)3(β2)2 and (α4)2(β2)3 nAChRs with similar potencies (*EC*_*50*_*s* of 0.34 ± 0.04 and 0.54 ± 0.02 μM, respectively) but has lower apparent efficacy at (α4)3(β2)2 than (α4)2(β2)3 nAChRs (*I*_*30 μM TC-2559*_ = 11 ± 2% and 152 ± 21% of current induced by 1 mM ACh, respectively) (Fig. 4). This is consistent with TC-2559 activating the receptor through the two α4:β2 ABS in both isoforms. The α4:α4 interface (Fig. 4*B*) is formed by residues from a (+) face of one α4 subunit and a (−) face of the adjacent α4 subunit. Amino acid residues forming the (−) face of α4 are unique and impose an additional layer of selectivity on agonist binding at the α4:α4 subunit interface (11, 35). Furthermore, amino acid substitutions at the α4 subunit (−) face have been shown to enable binding of agonists with larger molecular volumes at the α4:α4 subunit interface (34). Alanine substitution at α4H116 (α4H142 when amino acid numbering includes the signal peptide) within the α4 subunit (−) face allows TC-2559 to bind at the α4:α4 site (34) and results in increased TC-2559 efficacy (*E*_*max*_ of 182 ± 5% of current induced by 1 mM ACh *versus* ∼10% in WT) (Fig. 4*C*).

**Figure 5:**
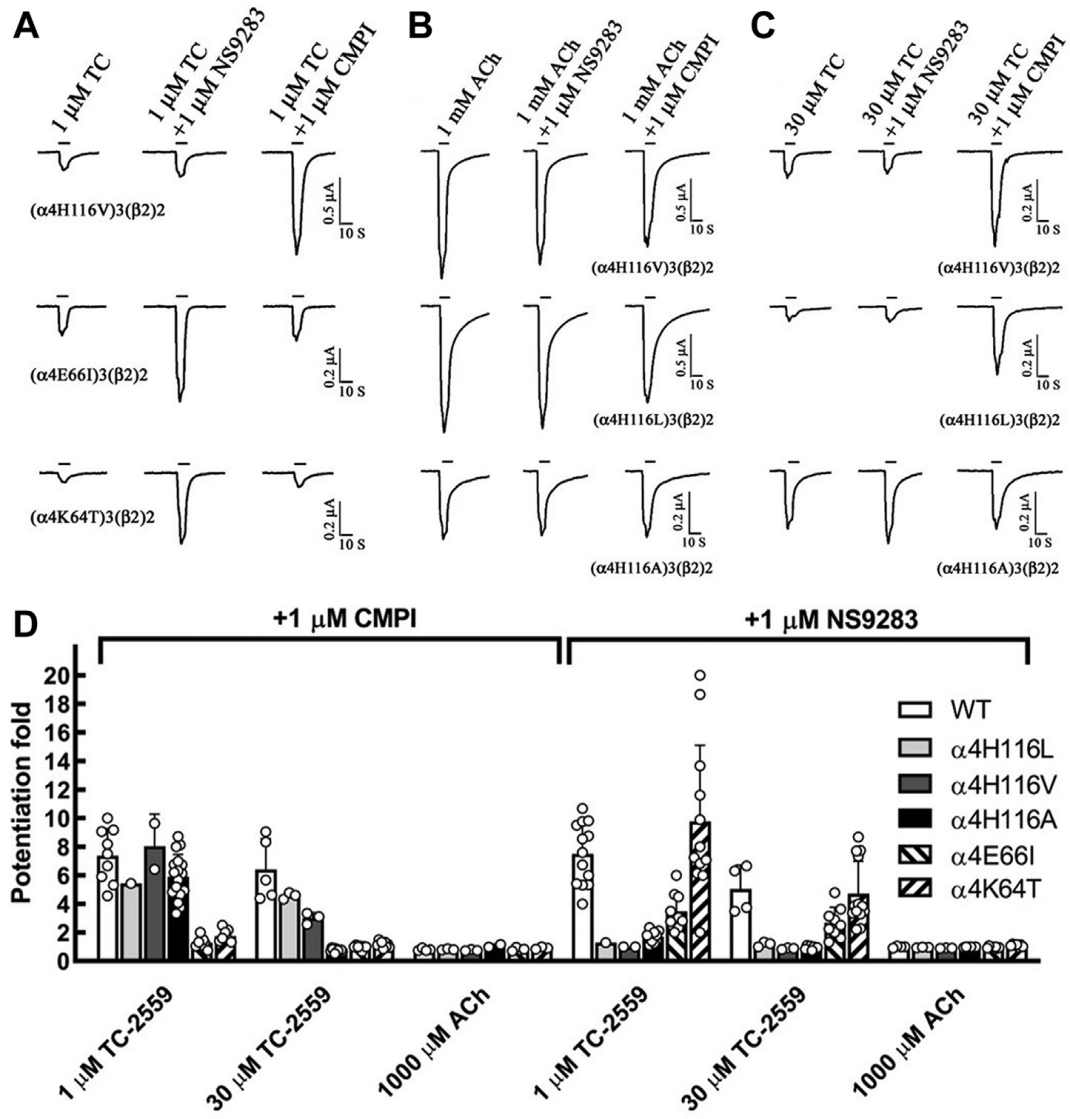
**Effects of mutations at the α4(−) interface on CMPI and NS9382 potentiation of TC-2559 induced currents of (α4)3(β2)2 nAChR.***A*–*C*, representative recordings of the whole-cell currents elicited by 10 s applications of agonist alone or with 1 μM of CMPI or NS9283. *D*, bar graph showing the peak currents elicited in response to agonist + 1 μM CMPI or agonist + 1 μM NS9283 normalized to the peak currents elicited by agonist alone applied in the same recording run. Statistical analysis of the effect of mutations on CMPI or NS9283 potentiation of current elicited by saturating concentrations of ACh or TC 2559 is shown in Table 1. ACh, acetylcholine; CMPI, 3-(2-chlorophenyl)-5-(5-methyl-1-(piperidin-4-yl)-1H-pyrrazol-4-yl)isoxazole; nAChR, nicotinic acetylcholine receptor.

We have previously shown that amino acid substitutions at positions α4K64 and α4E66, but not α4H116, significantly reduce CMPI-mediated potentiation of ACh-induced currents in the (α4)3(β2)2 nAChR (28). Similarly, CMPI did not potentiate TC-2559-induced current responses in (α4)3(β2)2 nAChR containing α4K64T or α4E66I (Fig. 5*A*). In addition, mutations at α4H116, which abolish potentiation by NS9283, did not affect CMPI potentiation of current responses of (α4)3(β2)2 nAChR induced by submaximal TC-2559 concentrations.

**Figure 6:**
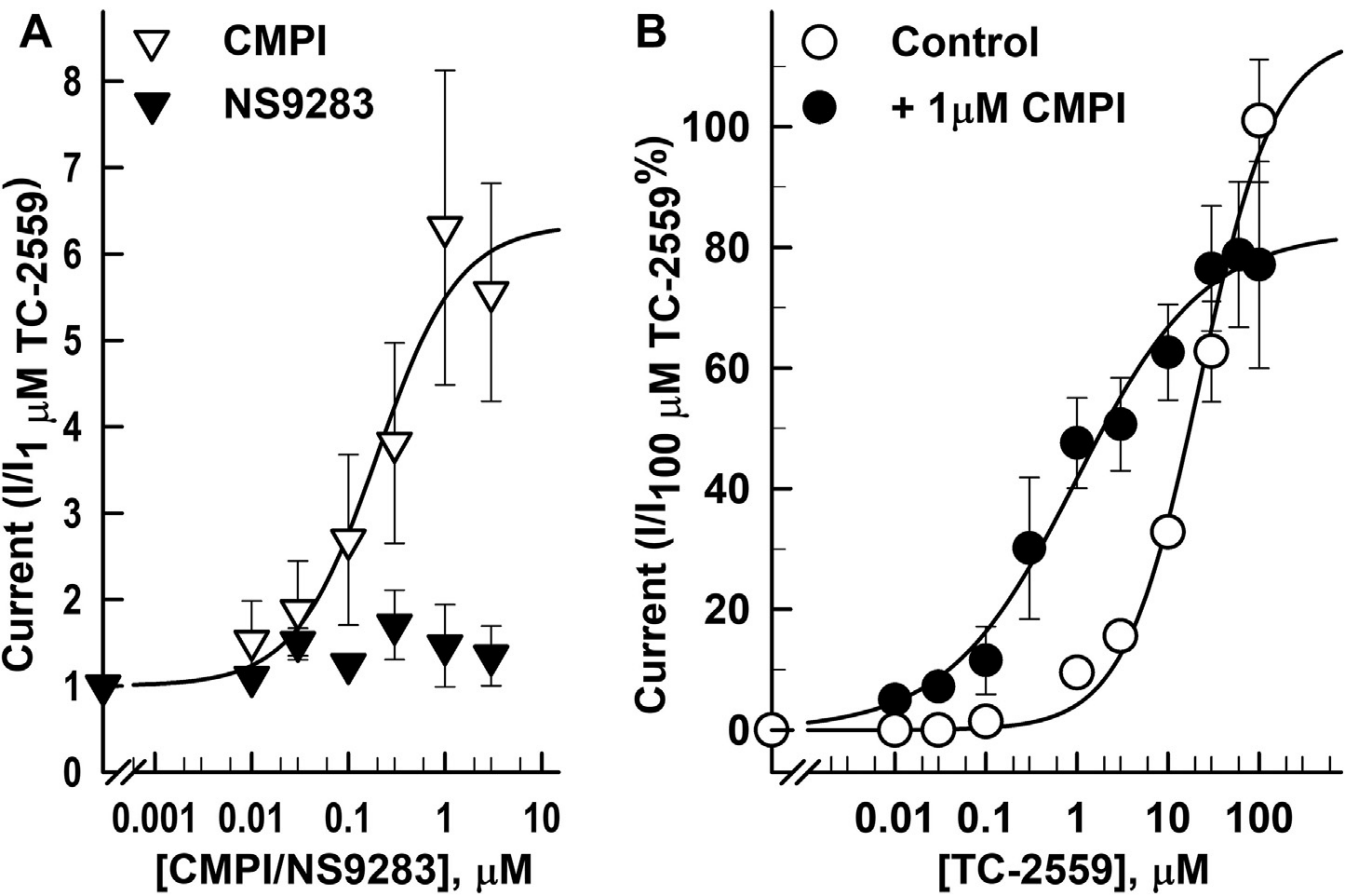
**Effect of coapplication of CMPI with TC-2559on (α4H116A)3(β2)2 nAChR.** The whole-cell currents elicited by 10 s applications of TC-2559 alone or in combination with CMPI or NS9283 from (α4)3(β2)2 nAChR containing α4H116A mutation. *A*, peak current was normalized to the peak current elicited by 1 μM TC-2559. *B*, peak current was normalized to the peak current elicited by 100 μM TC-2559. Each data point shown are the mean ± SD of data obtained from at least three oocytes. The data were fit to a single site model using Equation 1. CMPI, 3-(2-chlorophenyl)-5-(5-methyl-1-(piperidin-4-yl)-1H-pyrrazol-4-yl)isoxazole; nAChR, nicotinic acetylcholine receptor.

These results indicate that CMPI binds in the (α4)3(β2)2 nAChR to the same site and interacts with the same amino acid residues in the presence of ACh or TC-2559. The effect of amino acid substitutions at position α4H116 on CMPI potentiation of responses to saturating concentrations of TC-2559 was remarkable (Fig. 5, *B*–*D*). CMPI potentiation ratios of current induced by 30 μM TC-2559 in (α4)3(β2)2 nAChR containing amino acid substitution at α4H116 to leucine (α4H116L), valine (α4H116V), or alanine (α4H116A) were 4.58 ± 0.24, 3.01 ± 0.39, and 0.74 ± 0.13, respectively (Fig. 5*D*). Statistical analyses of the effects of these mutations on CMPI and NS9283 potentiation of responses to saturating concentrations of ACh and TC-2559 are shown in Table 1. The effect of 1 μM CMPI on current induced by 30 μM TC-2559 in (α4 H116L)3(β2)2, (α4H116V)3(β2)2, and (α4 H116A)3(β2)2 nAChRs was significantly different from that in WT (α4)3(β2)2 and not significantly different from no potentiation (Table 1). The simplest interpretation of this decline in CMPI-potentiation ratio is that reduction in the molecular volume of the aliphatic amino acid residue at position α4H116 (molecular volumes of L, V, and A are 166.7, 140.0, and 88.6 Å^3^, respectively) increases the affinity of TC-2559 at the α4:α4 interface, and thus at high TC-2559 concentrations, it reduces the ability of CMPI to bind at α4:α4 interface. Indeed, CMPI concentration-dependent potentiation and its effect on the TC-2559 concentration-response curve of (α4)3(β2)2 nAChR containing α4H116A substitution (Fig. 6) mirrored the effect of CMPI on ACh-induced current responses of WT (α4)3(β2)2 nAChR. CMPI potentiated TC-2559-induced current at (α4H116A)3(β2)2 nAChR with *EC*_*50*_ of 0.2 ± 0.1 μM and to *I*_*max*_ of 634 ± 77% of that of control. In the absence and presence of 1 μM CMPI, the *EC*_*50*_ of TC-2559 at (α4H116A)3(β2)2 nAChR were 22 ± 7 and 0.97 ± 0.32 μM, respectively. The *E*_*max*_ were 115 ± 14 and 83 ± 5% in the absence and presence of CMPI.

**Table 1:** Effect of mutations on CMPI and NS9283 potentiation of (α4)3(β2)2 nAChR current elicited by saturating concentrations of ACh and TC 2559

Agonist	Subunits combination	+1 μM CMPI			+1 μM NS9283		
		Ave ±SD	*p*_*versus control*_	*p*_*versus WT*_	Ave ± SD	*p*_*versus control*_	*p*_*versus WT*_
TC 2559 (30 μM)	(α4)3(β2)2 WT	6.41 ± 2.15	<0.001	-	5.05 ± 1.66	<0.001	-
	(α4K64T)3(β2)2	1.17 ± 0.21	1.000	<0.001	4.72 ± 2.27	<0.001	0.700
	(α4E66I)3(β2)2	0.99 ± 0.15	1.000	<0.001	2.68 ± 1.08	0.076	0.028
	(α4H116L)3(β2)2	4.58 ± 0.24	0.004	0.003	1.18 ± 0.17	1.000	0.006
	(α4H116V)3(β2)2	3.01 ± 0.39	0.518	<0.001	0.87 ± 0.06	1.000	0.004
	(α4H116A)3(β2)2	0.74 ± 0.13	1.000	<0.001	0.93 ± 0.12	1.000	<0.001
ACh (1 mM)	(α4)3(β2)2 WT	0.81 ± 0.15	0.969	-	1.00 ± 0.04	0.996	-
	(α4K64T)3(β2)2	0.91 ± 0.08	1.000	0.446	1.10 ± 0.08	1.000	0.036
	(α4E66I)3(β2)2	0.83 ± 0.10	1.000	0.931	0.95 ± 0.07	1.000	0.639
	(α4H116L)3(β2)2	0.82 ± 0.05	1.000	0.877	0.95 ± 0.03	1.000	0.631
	(α4H116V)3(β2)2	0.76 ± 0.05	1.000	0.827	0.90 ± 0.02	1.000	0.224
	(α4H116A)3(β2)2	1.07 ± 0.13	1.000	0.015	0.98 ± 0.03	1.000	0.799

The data from Figure 5*D* reporting current responses to agonist, agonist + 1 μM CMPI, and agonist + 1 μM NS9283 of oocytes expressing WT and mutants (α4)3(β2)2 nAChRs was analyzed using one-way ANOVA with multiple comparisons *versus* control group (Holm–Sidak method, SigmaPlot, Systat Software Inc). Shown in the table are the probability (*p*_*versus control*_) that calculated potentiation folds in the presence of 1 μM CMPI or 1 μM NS9283 differ from no potentiation and the probability (*p*_*versus WT*_) that calculated potentiation folds in the presence of 1 μM CMPI or 1 μM NS9283 for (α4)3(β2)2 nAChRs containing the indicated amino acid mutation differ from the calculated potentiation fold for WT (α4)3(β2)2 nAChRs.

### Analysis of (α4)3(β2)2 receptor activation in the framework of the Monod–Wyman–Changeux allosteric model

To gain mechanistic insight into receptor activation in the presence of CMPI, we analyzed the currents in the framework of a cyclic two-state (resting and active) allosteric activation model (31, 32, 33). In this model, channel opening is mediated by stabilization of the active state by an agonist that, by definition, has higher affinity to the active than resting state. In the presence of multiple active compounds, each drug independently and energetically additively contributes to the stabilization of the active state.

We commenced by converting the raw amplitudes of current responses to units of probability of being in the active state (P_A_ units). The constitutive probability of being active (P_A,constitutive_) and peak P_A_ of the response to 1 mM ACh in the (α4)3(β2)2 receptor were estimated by comparing the current responses to 100 μM mecamylamine, 1 mM ACh, and 1 mM ACh + 3 μM desformylflustrabromine (dFBr). The underlying assumption in this approach (36) is that the application of the blocker mecamylamine inhibits constitutively-active receptors, thereby revealing the current level corresponding to P_A_ ∼0, whereas the coapplication of ACh and the allosteric activator dFBr activates all receptors in the membrane and reveals the current level with P_A_ ∼1. The application of mecamylamine elicited outward current with the mean amplitude of 2.1 ± 0.5% of the absolute response to 1 mM ACh, whereas dFBr potentiated the peak response to 1 mM ACh to 278 ± 36% of control. From this, we estimate a P_A,constitutive_ of 0.00755 ± 0.00118 and a P_A,1 mM ACh_ of 0.36 ± 0.05.

**Figure 7:**
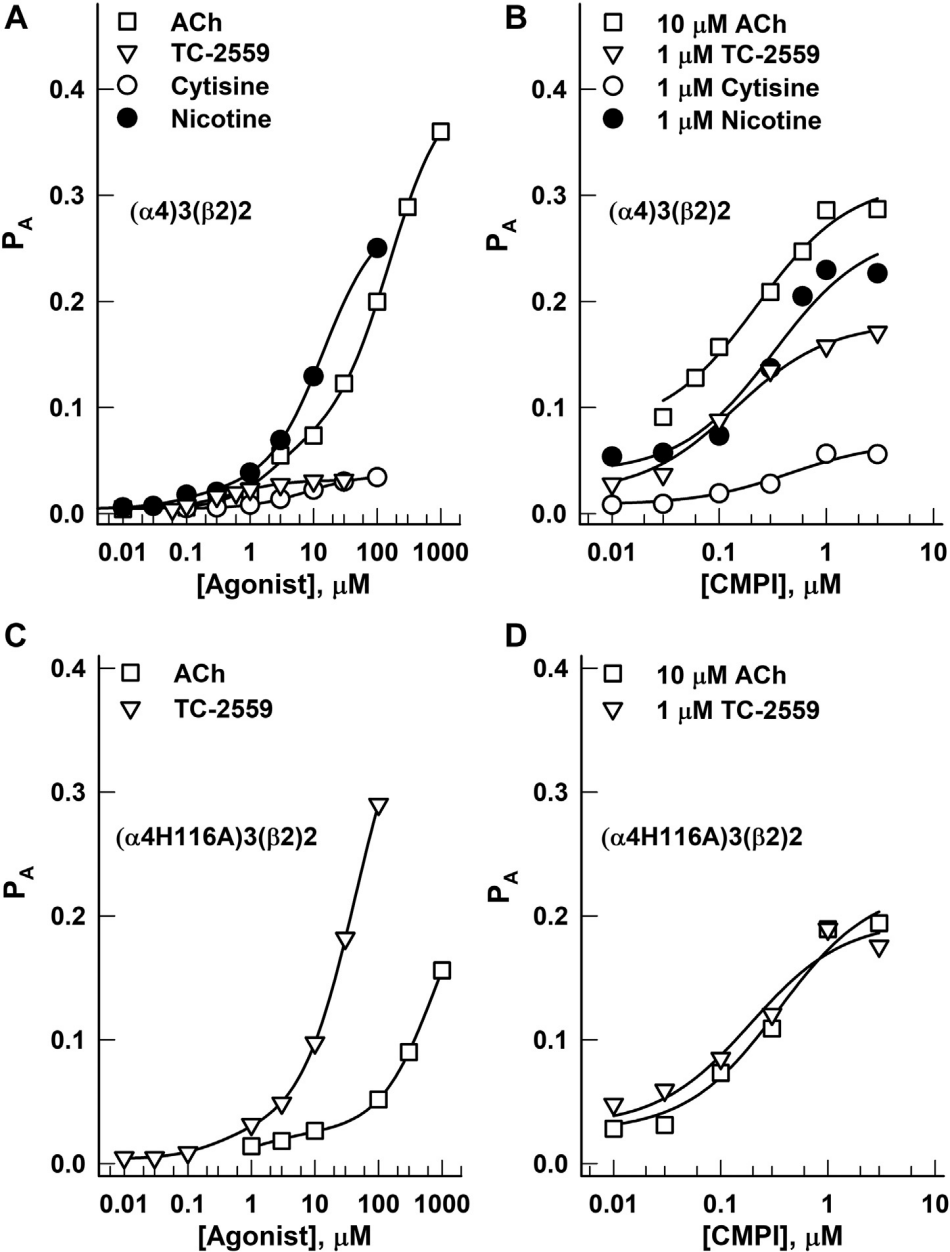
**Mechanistic analysis of receptor activity.** Peak current responses of WT (α4)3(β2)2 nAChR (*A* and *B*) and (α4)3(β2)2 nAChR containing α4H116A mutation (*C* and *D*) induced by applications of agonists (±CMPI) were converted to units of probability of being in the active state (P_A_) and analyzed using Equations (2), (3), (4), as described under Experimental procedures. The fitting parameters are listed in Table 2. ACh, acetylcholine; CMPI, 3-(2-chlorophenyl)-5-(5-methyl-1-(piperidin-4-yl)-1H-pyrrazol-4-yl)isoxazole; nAChR, nicotinic acetylcholine receptor.

The concentration-response relationships for ACh, cytisine, and nicotine were fitted to Equation 2. With L constrained to 220 (calculated as (1 − P_A,constitutive_)/P_A,constitutive_), the fitting yielded a K_R,ACh,α4:β2_ (equilibrium dissociation constant of ACh at the α4:β2 site in the resting receptor) of 1.32 ± 0.35 μM (best-fit parameter ±SD of the fit) and a *c*_ACh,α4:β2_ (ratio of the equilibrium dissociation constant of ACh at the α4:β2 site in the active receptor to K_R,ACh,α4:β2_) of 0.239 ± 0.014, and a K_R,ACh,α4:α4_ of 244 ± 25 μM and a *c*_ACh, α4:α4_ of 0.117 ± 0.013 (affinity and efficacy parameters of ACh, respectively, at the α4:α4 site). Thus, the binding of transmitter to the two α4:β2 sites and the single α4:α4 site contributes −1.69 and −1.27 kcal/mol, respectively, toward stabilization of the active state. Note that a lower value of *c* is associated with higher efficacy and that the single α4:α4 site in the presence of ACh contributes nearly as much as the combined two α4:β2 sites in free energy change. Fitting of the cytisine concentration-response curve to Equation 2 yielded a K_R,cytisine,α4:β2_ of 0.63 ± 1.21 μM, a *c*_cytisine,α4:β2_ of 0.875 ± 0.029, a K_R,cytisine,α4:α4_ of 8.3 ± 0.5 μM, and a *c*_cytisine,α4-α4_ of 0.155 ± 0.104. Fitting of the nicotine concentration-response curve gave a K_R,nicotine,α4:β2_ of 0.08 ± 0.05 μM, a *c*_nicotine,α4:β2_ of 0.421 ± 0.043, a K_R,nicotine,α4:α4_ of 19 ± 2 μM, and a *c*_nicotine,α4:α4_ of 0.065 ± 0.013. Thus, at the α4:β2 sites, ACh and cytisine have similar low μM affinities, whereas the affinity of nicotine is nearly ten-fold higher. At the α4:α4 site, all three agonists have significantly lower affinity. All three agonists act more efficaciously *via* the α4:α4 than a single α4:β2 site. The concentration-response curves are given in Figure 7, and the fitting results are summarized in Table 2.

**Table 2:** Summary of mechanistic analyses

Receptor	Ligand	Site	K_R_ (μM)	*c*	K_R,CMPI_ (μM)	*c*_CMPI_
(α4)3(β2)2 WT	ACh	α4:β2	1.32 ± 0.35	0.239 ± 0.014	-	-
		α4:α4	244 ± 25	0.117 ± 0.013	0.29 ± 0.06	0.145 ± 0.008
	Cytisine	α4:β2	0.63 ± 1.24	0.875 ± 0.029	-	-
		α4:α4	8.3 ± 0.5	0.155 ± 0.104	0.43 ± 0.17	0.074 ± 0.009
	Nicotine	α4:β2	0.08 ± 0.05	0.421 ± 0.043	-	-
		α4:α4	19 ± 2	0.065 ± 0.013	0.40 ± 0.10	0.065 ± 0.007
	TC-2559	α4:β2	0.33 ± 0.05	0.371 ± 0.006	-	-
		α4:α4	-	-	0.17 ± 0.03	0.090 ± 0.004
(α4H116A)3(β2)2	ACh	α4:β2	0.86 ± 0.24	0.365 ± 0.014	-	-
		α4:α4	1006 ± 116	0.076 ± 0.005	0.44 ± 0.12	0.082 ± 0.007
	TC-2559	α4:β2	0.21 ± 0.05	0.337 ± 0.013	-	-
		α4:α4	70 ± 3	0.046 ± 0.003	0.23 ± 0.08	0.099 ± 0.009

The table gives the fitted equilibrium dissociation constants for ACh, cytisine, nicotine, and TC-2559 in the resting receptor (K_R_) and the ratios of the equilibrium dissociation constants for the agonist in the active receptor to that in the resting receptor (*c*), at the α4:β2 and α4:α4 sites, for WT and α4(H116A) mutant receptors. The K_R, CMPI,_ and *c*_*CMPI*_ show the same for the PAM CMPI at the α4:α4 site, measured on the background of activity elicited by ACh, cytisine, nicotine, or TC-2559.

TC-2559 only interacts with the agonist-binding sites at the α4:β2 interface (30, 34). The concentration-response curve for TC-2559 was fitted to Equation 3, which describes a model with a single class of binding sites. The fitting yielded a K_R,TC-2559,α4:β2_ of 0.33 ± 0.05 μM and a *c*_TC-2559,α4:β2_ of 0.371 ± 0.006. Thus, TC-2559 has similar to ACh affinity and efficacy at the α4:β2 sites, and its overall lower gating efficacy (Fig. 7) is explained by its inability to contribute to channel activation *via* the α4:α4 site.

CMPI, which binds only to the α4:α4 interface, is a very weak direct activator of the α4β2 receptor (28). We therefore estimated the affinity and gating parameters for CMPI by measuring its effect on the background of activity elicited by a low concentration of TC-2559. Because TC-2559 only interacts with the agonist-binding sites at the α4:β2 interface (30, 34), its activating effect was reflected in a reduced value of L in Equation 3, calculated as (1 − P_A,TC-2559_)/P_A,TC-2559_. Using a dataset obtained in the presence of 1 μM TC-2559 and 0.01 to 3 μM CMPI, we estimate a K_R,CMPI,α4:α4_ of 0.17 ± 0.03 μM and a *c*_CMPI,α4:α4_ of 0.090 ± 0.004. The binding of CMPI to the α4:α4 interface contributes −1.42 kcal/mol of free energy change toward stabilization of the active state. CMPI is thus as efficacious as ACh at the α4:α4 site, and its overall low efficacy is accounted for by its single binding site.

Additional estimates of the properties of CMPI were obtained by measuring receptor activation by CMPI on the background of activity elicited by 10 μM TC-2559, 10 μM ACh, 1 μM cytisine, or 1 μM nicotine. Fitting the CMPI concentration-response data obtained in the presence of 10 μM TC-2559 to Equation 2 yielded a K_R,CMPI,α4:α4_ of 0.09 ± 0.02 μM and a *c*_CMPI,α4:α4_ of 0.129 ± 0.009. The data for the combinations of CMPI with ACh, cytisine, or nicotine were analyzed using Equation 4, which describes a model in which CMPI competes with ACh, cytisine, or nicotine, respectively, at the α4:α4 site. Fitting the 10 μM ACh + CMPI data to Equation 4 gave a K_R,CMPI,α4:α4_ of 0.29 ± 0.06 μM and a *c*_CMPI,α4:α4_ of 0.145 ± 0.008, and the combination of 1 μM cytisine + CMPI yielded a K_R,CMPI,α4:α4_ of 0.43 ± 0.17 μM and a *c*_CMPI,α4:α4_ of 0.074 ± 0.009. From the combination of 1 μM nicotine + CMPI, we estimate a K_R,CMPI,α4:α4_ of 0.40 ± 0.10 μM and a *c*_CMPI,α4:α4_ of 0.065 ± 0.007.

Thus, the five approaches generated estimates that were within a factor of ∼5 for K_R,CMPI,α4:α4_ (range: 0.09–0.43 μM) and ∼2 for *c*_CMPI,α4:α4_ (range: 0.065–0.145). We do not know the reason for the rather large range of estimates for K_R,CMPI,α4:α4_, but allosteric coupling between the α4:β2 and α4:α4 sites and additional effects through as yet unidentified sites are some possibilities to be tested in future work. From the five approaches, the calculated mean K_R,CMPI,α4:α4_ is 0.28 ± 0.15 μM and the mean *c*_CMPI,α4:α4_ 0.101 ± 0.035.

## Discussion

Prior pharmacological studies have identified multiple ligand-recognition sites within the extracellular and transmembrane domains of the (α4)3(β2)2 nAChR including the “canonical” α4:β2 and the “noncanonical” α4:α4 ABS. The functional response (*i.e.*, channel gating) to ligand occupancy at these recognition sites depends on which site(s) is occupied, the number of site(s) occupied, the allosteric coupling of the occupied site with channel gating, and the intrinsic activity of the ligand at the site(s) it occupies. The small molecule agonists including ACh, nicotine, and cytisine bind with high affinity to the α4:β2 ABS and with a much lower affinity to the α4:α4 ACh binding (11, 34). In contrast, larger agonists with a maximum length of >7.5 Å and an accessible surface area of >300 Å (*e.g.*, TC-2559) only bind to the α4:β2 ABS (30, 34). Compounds that bind with high affinity at the α4:α4 ACh-binding site (*e.g.*, CMPI and NS9283) have been identified. CMPI was introduced as the PAM of (α4)3(β2)2 nAChR as it enhanced channel responses when coapplied with ACh (12, 28). Here, we have characterized the effect of coapplication of CMPI with a series of agonists with different intrinsic activities at the (α4)3(β2)2 nAChR and different binding properties at the α4:α4 ACh-binding site. CMPI potentiated (α4)3(β2)2 nAChR responses to subsaturating concentrations of all tested agonists, independent of their intrinsic activity or α4:α4 binding properties. In contrast, CMPI potentiation of (α4)3(β2)2 nAChR responses induced by a saturating agonist was dependent on the ability of the agonist to bind at the α4:α4 ACh-binding site. CMPI enhanced the response to saturating TC-2559 in the WT receptor where TC-2559 only binds to the α4:β2 ACh-binding site, but not in the receptor containing the α4H116A mutation that enables the binding of TC-2559 to the α4:α4 ACh-binding site. Analysis of the findings in the framework of the MWC model indicates that CMPI binds at the α4:α4 interface with higher affinity than ACh, cytisine, or nicotine whereas its gating efficacy at the α4:α4 site is equivalent to that of ACh, cytisine, or nicotine. Therefore, the weak direct activating effect of CMPI is accounted for by a single-binding site mediating its action.

We estimated the binding and gating properties of CMPI in the presence of TC-2259 (applied at two concentrations), ACh, cytisine, or nicotine. The different approaches yielded estimates that were within a factor of ∼5 for K_R,CMPI_ and ∼2 for *c*_CMPI_. One potential explanation to this relatively large range of estimates is allosteric coupling between the α4:β2 and α4:α4 sites. This possibility could be tested by comparing receptor activation, individually and in combination, by agents selectively interacting with the α4:β2 and α4:α4 sites. At present, however, we lack the tools to independently measure activation produced by selective occupation of the α4:α4 site.

Another possible explanation is that one or more of the drugs act through other sites or mechanisms that are not incorporated into the MWC model. Several orthosteric agonists including nicotine show reduced peak response at high agonist concentrations, possibly a result of open-channel blocking mechanism (37). This inhibitory effect, which is not accounted for by our model, may be expected to predominantly affect the fitted K_R_ and *c* at the low-affinity α4:α4 site. The inhibitory effect is, however, minimized when CMPI-elicited currents are recorded in the presence of low concentration (1 μM) of nicotine. Finally and, what we consider, most plausible is that the differences in estimated K_R,CMPI_ and *c*_CMPI_ reflect simple experimental imprecision and variability in receptor behavior. This is supported by the finding that a change in the concentration of background TC-2559 from 1 to 10 μM generates an almost two-fold change in estimated K_R,CMPI_.

The estimated activation parameters presented here are dependent on the accurate measurement of the constitutive P_A_ of the α4β2 receptor and its peak P_A_ in the presence of ACh. We used mecamylamine to block constitutively active receptors and reveal the current level corresponding to a P_A_ of 0. Mecamylamine is a nonselective, allosteric antagonist of the nAChR with IC_50_s in the submicromolar range (38). It acts by blocking open receptors (39). We measured the effect of 100 μM mecamylamine on holding current to estimate the current level at P_A_ of 0. Underestimation of the effect of mecamylamine on holding current would lead to underestimated P_A,constitutive_ and an overestimated L (Equation 2). This would introduce an error in the estimated values of *c* for ACh, cytisine, TC-2559, and nicotine. The extent of error can be calculated from the relationship L_true_ × *c*_true_^2^ = L_estimated_ × *c*_estimated_^2^. An underestimated P_A,constitutive_ is not expected to lead to a meaningful error in the activation parameters for CMPI or the peak P_A,ACh_ values.

The peak P_A,ACh_ (0.36) was estimated by normalizing the peak response to 1 mM ACh to that in the presence of 1 mM ACh + 3 μM dFBr. dFBr is a brominated alkaloid, originally isolated from the marine bryozoan *Flustra foliacea* (40) that selectively and allosterically potentiates the α4β2 nAChR (41). In our hands, 3 μM dFBr almost tripled the peak response to 1 mM ACh. We have assumed that the response to ACh + dFBr has a peak P_A_ indistinguishable from 1. An underestimated potentiating effect of dFBr would lead to overestimated peak P_A_ for ACh. This, in turn, would lead to proportional errors in estimated *c* for each of the tested ligands. However, the estimated relative contributions made by α4:β2 and α4:α4 sites would remain unaffected. The previous studies have reported a peak P_A,ACh_ of 0.5 to >0.8 in the (α4)3(β2)2 nAChR (30, 42). Indurthi *et al.* (30) used an approach similar to ours, observing doubling of the peak response to 1 mM ACh in the presence of the allosteric modulator NS206, whereas Li and Steinbach (42) employed nonstationary noise analysis on human embryonic kidney cells stably expressing the α4β2 receptor.

A previous study reported that pharmacological elimination of the α4:α4 site in the (α4)3(β2)2 nAChR reduced the subsequent response to saturating ACh to ∼40% of the control response (34). The fraction of the high-affinity component in the ACh concentration-response relationship remains at ∼15% even when the α4 subunit is expressed in excess (11), indicating that occupation of the α4:β2 sites by ACh generates a functional response that is 15 to 40% of the response to saturating ACh in the (α4)3(β2)2 receptor. This is in good agreement with the data presented here. Using the K_R,ACh_ and *c*_ACh_ values in Table 2, we calculate, using Equation 2, that occupation of the two α4:β2 sites in the (α4)3(β2)2 receptor with ACh generates a peak P_A_ of 0.072, whereas occupation of the two α4:β2 sites and the single α4:α4 site with ACh generates a peak P_A_ of 0.36. We emphasize that in either case, ACh should be considered a partial agonist of the (α4)3(β2)2 nAChR given its relatively low maximal P_A_.

In sum, we report here that ACh binds with high affinity (K_R,ACh,α4:β2_ = 1.32 μM) to the α4:β2 agonist-binding sites where it acts with relatively low efficacy (*c*_ACh,α4:β2_ = 0.239; ΔG_gating,total_ = −1.69 kcal/mol or −0.84 kcal/mol per site). ACh binds with low affinity (K_R,ACh,α4:α4_ = 244 μM) to the α4:α4 site where it acts with relatively high efficacy (c_ACh, α4:α4_= 0.117; ΔG_gating_ = −1.27 kcal/mol). The nicotinic receptor PAM CMPI has high affinity to the α4:α4 site (K_R,CMPI,α4:α4_ = 0.28 μM) and efficacy comparable to that of ACh (*c*_CMPI,α4:α4_ 0.101; ΔG_gating_ = −1.35 kcal/mol). CMPI enhances channel-gating activation triggered by ACh occupancy at the α4:β2 agonist-binding sites by binding to the α4:α4 subunit interface which becomes occupied by ACh only at high concentrations. Overall, these results indicate that exposure to agonists targeting the α4:α4 binding site in the (α4)3(β2)2 nAChR is expected to increase the efficacy of the transmitter ACh, that may be therapeutically beneficial in conditions associated with decline in the output of nAChR in the brain.

## Experimental procedures

### Materials

Acetylcholine chloride was purchased from Sigma-Aldrich. Other ligands of the nAChR (CMPI, NS9283, dFBr, TC-2559, cytisine, and mecamylamine) were from Tocris Bioscience R&D. Collagenase type 2 was from Worthington Biomedical. The stock solutions were prepared for ACh (1 M in water) and other nAChR ligands (10 mM in water or DMSO) and stored in aliquots at −20 °C until used. The final working solutions were prepared in recording buffer on the day of experiments.

### Expression of (α4)3(β2)2 and (α4)2(β2)3 nAChRs in *Xenopus* oocytes

Oocytes-positive female *Xenopus laevis* were purchased from NASCO, and all procedures were performed according to an animal use protocol approved by the Institutional Animals Care and Use Committee of The University of Texas Health Science Center at Tyler. Ovarian lobules were surgically harvested, treated with collagenase type 2, and Stage V and VI oocytes were visually selected and maintained at 18 °C in modified ND96-gentamicin buffer (96 mM NaCl, 2 mM KCl, 1.8 mM CaCl_2_, 1 mM MgCl_2_, 5 mM Hepes, and 50 μg/ml gentamicin, pH 7.6).

pSP64 Poly(A) plasmids with cDNA encoding for human α4 or β2 nAChR subunit were used to prepare cRNA transcripts suitable for oocyte expression. The plasmids were linearized with AseI (hα4) and PvuII (hβ2), then cRNA transcripts were prepared *in vitro* using mMESSAGE mMACHINE high yield-capped RNA transcription kits (Ambion, Thermo Fisher Scientific), purified on NucAway Spin column (Invitrogen, Thermo Fisher Scientific), and stored at −80 °C until used. Point mutations within the plasmid encoding the α4 nAChR subunit were introduced using QuikChange II Site-Directed Mutagenesis Kit (Agilent Technologies), as described previously (28). To generate amino acid substitutions (K64T, E66I, H116V, H116L, and H116A), two custom-designed complementary oligos containing the desired mutation were used (Integrated DNA Technologies). The forward primers were as the following with the codon for mutated amino acids are underlined and nucleotide(s) changes are bolded and italicized:

α4K64T, 5′-G AAC GTA TGG GTG A***CA*** CAG GAG TGG CAC-3′; α4E66I, 5′- C GTA TGG GTG AAG CAG ***ATC*** TGG CAC GAC-3′; α4H116V, 5′-CAC CTG ACC AAG GCC ***GTA*** CTG TTC CAT G-3′; α4H116L, 5′-CTG ACC AAG GCC C***T***C CTG TTC CAT GAC-3′; and α4H116A, 5′-CTG ACC AAG GCC ***GC***C CTG TTC CAT GAC-3′.

The oocytes were injected with 10 to 50 ng of a cRNA mix containing α4 (WT or mutant) and β2 RNAs at ratios of 8:1(α4:β) or 1:8 (α4:β2) to bias expression toward (α4)3(β2)2 or (α4)2(β2)3 nAChRs, respectively. The injection of oocytes with RNAs mixture containing four folds or higher α4 RNA than β2 RNA have been established to express receptor population that is made up of three α4 and two β2 subunits (9, 12, 28, 43, 44, 45).

### Two-electrode voltage-clamp recordings

Two-electrode voltage-clamp recordings of ACh- or TC-2559 induced responses of *Xenopus* oocytes were performed, as described in (28). 24 to 72 h after cRNA injection, *Xenopus* oocytes were placed in a custom-made recording chamber that is connected to an eight-channel automated perfusion system (Warner Instruments) and perfused with recording buffer (100 mM NaCl, 2 mM KCl, 1 mM CaCl_2_, 0.8 mM MgCl_2_, 1 mM EGTA, and 10 mM Hepes, pH 7.5). Unless otherwise specified in figure legends, each recording run included several drug applications (10 s of an agonist with or without CMPI or NS9283) separated by 3 to 4 min buffer wash intervals. Between recording runs, the oocytes were washed with recording buffer for at last 5 min. The oocytes were voltage-clamped at −50 mV using Oocyte Clamp OC-725B (Warner Instruments). The currents were digitized using Digidata 1550A (Axon Instruments, Molecular Devices), and the peak currents were quantified using pCLAMP 10 (Axon Instruments), then normalized and analyzed using Excel 2010 (Microsoft) and SigmaPlot 11.0 (Systat Software). For NS9283 and CMPI potentiation of agonist-induced responses, the peak currents were normalized to current elicited by agonist alone applied within the same recording run. For the effect of coapplication of 1 μM NS9283 or CMPI on agonist concentration-response curve, the peak currents were normalized to current elicited by saturating concentration of agonist applied within the same recording run. Mean ± SD of N oocytes were plotted and fit to the following equation:

(1) $${\text{I}}_{\text{X}}={\text{I}}_{0}+\frac{{\text{I}}_{\text{max}}}{1+{\left(\frac{EC_{50}}{\text{X}}\right)}^{\text{h}}}$$

where I_x_ is the normalized agonist-induced current in the presence of NS9283 or CMPI at concentration x, I_max_ is the maximum potentiation of current, h is the Hill coefficient, and *EC*_*50*_ is the of NS9283 or CMPI concentration producing 50% of maximal potentiation. I_0_ = 100 for NS9283 and CMPI potentiation of agonist-induced responses and I_0_ = 0 for agonist concentration-response experiments. The best-fit values for I_max_ and *EC*_*50*_ ± SD are presented.

For the enhancement of agonist-induced currents by 1 μM CMPI or NS9283 (data in Figs. 1*C* and 5*D*), the probability (*P*) that the calculated potentiation fold differ from no potentiation (potentiation fold = 1) or from WT (α4)3(β2)2 nAChR (potentiation fold of 6.21 and 5.05 for CMPI or NS9283, respectively) was analyzed using one-way analysis of variance with Holm–Sidak post hoc test (SigmaPlot, Systat Software Inc) and reported in the legend for Figure 1*C* and Table 1.

### Mechanistic analysis

Further analysis of electrophysiological data was conducted in the framework of the two-state concerted transition model, adapted from the MWC cyclic model originally used to describe enzyme function (31, 32, 33). The raw peak amplitudes of current responses were converted to units of probability of being in the active state (P_A_). We used a multi-step approach where the peak P_A_ to 1 mM ACh was estimated through normalization to the peak response to 1 mM ACh +3 μM dFBr. Additional normalization was carried out by comparing responses to various agonists or agonist combinations to the peak response to 1 mM ACh in the same set of cells. The P_A_ of constitutive activity (P_A,constitutive_) was estimated by comparing the effects of 100 μM mecamylamine and 1 mM ACh on the holding current.

The (α4)3(β2)2 receptor contains two binding sites for ACh at the α4:β2 intersubunit interface and one site at the α4:α4 interface (11). The same set of sites has also been shown to mediate receptor activation by the alkaloid cytisine (34). The concentration-response curves for ACh, cytisine, and nicotine were fitted to the state function:

(2) $${\text{P}}_{\text{A}}=\frac{1}{1+\text{L}{\left[\frac{1+\left[\text{agonist}\right]{\text{/K}}_{\text{R,agonist,α}4\text{:β}2}}{1+\left[\text{agonist}\right]\text{/}\left({\text{K}}_{\text{R,agonist,α}4\text{:β}2}c_{\text{agonist,α}4\text{:β}2}\right)}\right]}^{{\text{N}}_{\text{α}4\text{:β}2}}{\left[\frac{1+\left[\text{agonist}\right]{\text{/K}}_{\text{R,agonist,α}4\text{:α}4}}{1+\left[\text{agonist}\right]\text{/}\left({\text{K}}_{\text{R,agonist,α}4\text{:α}4}c_{\text{agonist,α}4\text{:α}4}\right)}\right]}^{{\text{N}}_{\text{α}4\text{:α}4}}}$$

where L indicates the level of background activity in the absence of agonist and is calculated as (1 − P_A,constitutive_)/P_A,constitutive_, [agonist] is the concentration of ACh, cytisine, or nicotine, K_R,agonist,α4:β2_ and K_R,agonist,α4:α4_ are the equilibrium dissociation constant for the agonist in the resting receptor at the α4-β2 or α4-α4 sites, respectively, and *c*_agonist,α4:β2_ and *c*_agonist,α4:α4_ are the ratios of the equilibrium dissociation constants for the agonist in the active receptor to that in the resting receptor. The numbers of α4:β2 and α4:α4 binding sites (N_α4:β2_ and N_α4:α4_) were constrained to 2 and 1, respectively.

TC-2559 activates the (α4)3(β2)2 receptor by binding to the two sites at the α4:β2 interface. The concentration-response curve for TC-2559 was fitted to the following equation:

(3) $${\text{P}}_{\text{A}}=\frac{1}{1+\text{L}{\left[\frac{1+\left[\text{TC}-2559\right]{\text{/K}}_{\text{R,TC}-2559\text{,α}4\text{:β}2}}{1+\left[\text{TC}-2559\right]\text{/}\left({\text{K}}_{\text{R,TC}-2559\text{,α}4\text{:β}2}c_{\text{TC}-2559\text{,α}4\text{:β}2}\right)}\right]}^{{\text{N}}_{\text{α}4\text{:β}2}}}$$

The terms are as described above.

CMPI interacts with the agonist-binding site at the α4-α4 interface. Because it is a weak agonist, its affinity and gating properties at the α4-α4 site were estimated by coapplying CMPI with a fixed, low concentration of TC-2559. The concentration-response data were analyzed using Equation 3, with the value of L modified to reflect receptor activation by TC-2559, and the affinity and efficacy terms in the equation reflecting the values for CMPI. The number of binding sites for CMPI in Equation 3 was constrained to 1.

For the combinations of ACh, cytisine or nicotine, and CMPI we assumed that ACh, cytisine, and nicotine are the sole ligands at the two α4:β2 sites, whereas CMPI competes with ACh, cytisine, or nicotine at the α4-α4 site. The concentration-response curves for the combinations of ACh, cytisine or nicotine, plus CMPI were fitted to the following equation:

(4) $${\text{P}}_{\text{A}}=\frac{1}{1+\text{L}{\left[\frac{1+\left[\text{agonist}\right]{\text{/K}}_{\text{R,agonist,α}4\text{:β}2}}{1+\left[\text{agonist}\right]\text{/}\left({\text{K}}_{\text{R,agonist,α}4\text{:β}2}c_{\text{agonist,α}4\text{:β}2}\right)}\right]}^{{\text{N}}_{\text{α}4\text{:β}2}}{\left[\frac{1+\left[\text{agonist}\right]{\text{/K}}_{\text{R,agonist,α}4\text{:α}4}+\left[\text{CMPI}\right]{\text{/K}}_{\text{R,CMPI,α}4\text{:α}4}}{1+\left[\text{agonist}\right]\text{/}\left({\text{K}}_{\text{R,agonist,α}4\text{:α}4}c_{\text{agonist,α}4\text{:α}4}\right)+\left[\text{CMPI}\right]\text{/}\left({\text{K}}_{\text{R,CMPI,α}4\text{:α}4}c_{\text{CMPI,α}4\text{:α}4}\right)}\right]}^{{\text{N}}_{\text{α}4\text{:α}4}}}$$

where K_R,CMPI_ is the equilibrium dissociation constant for CMPI in the resting receptor at the α4:α4 site, *c*_CMPI_ is the ratio of the equilibrium dissociation constants for CMPI in the active receptor to K_R,CMPI_, and other terms are as described above.

Curve fitting was done using Origin 2020 (OriginLab Corp). The results are reported as best-fit parameter ±SD of the fit. All data are included in the analysis.

## Data availability

All data are contained in the article.

## Conflict of interest

The authors declare that they have no conflicts of interest with the contents of this article.

## References

[1] Gotti C., Clementi F. (2004). Neuronal nicotinic receptors: From structure to pathology. Prog. Neurobiol..

[2] Dani J.A., Bertrand D. (2007). Nicotinic acetylcholine receptors and nicotinic cholinergic mechanisms of the central nervous system. Annu. Rev. Pharmacol. Toxicol..

[3] Hurst R., Rollema H., Bertrand D. (2013). Nicotinic acetylcholine receptors: From basic science to therapeutics. Pharmacol. Ther..

[4] Jensen A.A., Frolund B., Liljefors T., Krogsgaard-Larsen P. (2005). Neuronal nicotinic acetylcholine receptors: Structural revelations, target identifications, and therapeutic inspirations. J. Med. Chem..

[5] Taly A., Corringer P.J., Guedin D., Lestage P., Changeux J.P. (2009). Nicotinic receptors: Allosteric transitions and therapeutic targets in the nervous system. Nat. Rev. Drug Discov..

[6] Mohamed T.S., Jayakar S.S., Hamouda A.K. (2015). Orthosteric and allosteric ligands of nicotinic acetylcholine receptors for smoking cessation. Front. Mol. Neurosci..

[7] Changeux J.P. (2010). Nicotine addiction and nicotinic receptors: Lessons from genetically modified mice. Nat. Rev. Neurosci..

[8] Changeux J.P. (2010). Allosteric receptors: From electric organ to cognition. Annu. Rev. Pharmacol. Toxicol..

[9] Nelson M.E., Kuryatov A., Choi C.H., Zhou Y., Lindstrom J. (2003). Alternate stoichiometries of alpha4beta2 nicotinic acetylcholine receptors. Mol. Pharmacol..

[10] Moroni M., Bermudez I. (2006). Stoichiometry and pharmacology of two human alpha4beta2 nicotinic receptor types. J. Mol. Neurosci..

[11] Harpsoe K., Ahring P.K., Christensen J.K., Jensen M.L., Peters D., Balle T. (2011). Unraveling the high- and low-sensitivity agonist responses of nicotinic acetylcholine receptors. J. Neurosci..

[12] Timmermann D.B., Sandager-Nielsen K., Dyhring T., Smith M., Jacobsen A.M., Nielsen E.O., Grunnet M., Christensen J.K., Peters D., Kohlhaas K., Olsen G.M., Ahring P.K. (2012). Augmentation of cognitive function by NS9283, a stoichiometry-dependent positive allosteric modulator of alpha2- and alpha4-containing nicotinic acetylcholine receptors. Br. J. Pharmacol..

[13] Benallegue N., Mazzaferro S., Alcaino C., Bermudez I. (2013). The additional ACh binding site at the alpha4(+)/alpha4(-) interface of the (alpha4beta2)2alpha4 nicotinic ACh receptor contributes to desensitization. Br. J. Pharmacol..

[14] Carbone A.L., Moroni M., Groot-Kormelink P.J., Bermudez I. (2009). Pentameric concatenated (alpha4)(2)(beta2)(3) and (alpha4)(3)(beta2)(2) nicotinic acetylcholine receptors: Subunit arrangement determines functional expression. Br. J. Pharmacol..

[15] Hamouda A.K., Deba F., Wang Z.J., Cohen J.B. (2016). Photolabeling a nicotinic acetylcholine receptor (nAChR) with an (alpha4)3(beta2)2 nAChR-selective positive allosteric modulator. Mol. Pharmacol..

[16] Marks M.J., Whiteaker P., Calcaterra J., Stitzel J.A., Bullock A.E., Grady S.R., Picciotto M.R., Changeux J.P., Collins A.C. (1999). Two pharmacologically distinct components of nicotinic receptor-mediated rubidium efflux in mouse brain require the beta2 subunit. J. Pharmacol. Exp. Ther..

[17] DeDominicis K.E., Sahibzada N., Olson T.T., Xiao Y., Wolfe B.B., Kellar K.J., Yasuda R.P. (2017). The (alpha4)3(beta2)2 stoichiometry of the nicotinic acetylcholine receptor predominates in the rat motor cortex. Mol. Pharmacol..

[18] Hamouda A.K., Bautista M.R., Akinola L.S., Alkhlaif Y., Jackson A., Carper M., Toma W.B., Garai S., Chen Y.C., Thakur G.A., Fowler C.D., Damaj M.I. (2021). Potentiation of (alpha4)2(beta2)3, but not (alpha4)3(beta2)2, nicotinic acetylcholine receptors reduces nicotine self-administration and withdrawal symptoms. Neuropharmacology.

[19] Lester H.A., Xiao C., Srinivasan R., Son C.D., Miwa J., Pantoja R., Banghart M.R., Dougherty D.A., Goate A.M., Wang J.C. (2009). Nicotine is a selective pharmacological chaperone of acetylcholine receptor number and stoichiometry. Implications for drug discovery. AAPS J..

[20] Srinivasan R., Pantoja R., Moss F.J., Mackey E.D., Son C.D., Miwa J., Lester H.A. (2011). Nicotine up-regulates alpha4beta2 nicotinic receptors and ER exit sites via stoichiometry-dependent chaperoning. J. Gen. Physiol..

[21] Vallejo Y.F., Buisson B., Bertrand D., Green W.N. (2005). Chronic nicotine exposure upregulates nicotinic receptors by a novel mechanism. J. Neurosci..

[22] Kim J.S., Padnya A., Weltzin M., Edmonds B.W., Schulte M.K., Glennon R.A. (2007). Synthesis of desformylflustrabromine and its evaluation as an alpha4beta2 and alpha7 nACh receptor modulator. Bioorg. Med. Chem. Lett..

[23] Albrecht B.K., Berry V., Boezio A.A., Cao L., Clarkin K., Guo W., Harmange J.C., Hierl M., Huang L., Janosky B., Knop J., Malmberg A., McDermott J.S., Nguyen H.Q., Springer S.K. (2008). Discovery and optimization of substituted piperidines as potent, selective, CNS-penetrant alpha4beta2 nicotinic acetylcholine receptor potentiators. Bioorg. Med. Chem. Lett..

[24] Olsen J.A., Ahring P.K., Kastrup J.S., Gajhede M., Balle T. (2014). Structural and functional studies of the modulator NS9283 reveal agonist-like mechanism of action at alpha4beta2 nicotinic acetylcholine receptors. J. Biol. Chem..

[25] Springer S.K., Woodin K.S., Berry V., Boezio A.A., Cao L., Clarkin K., Harmange J.C., Hierl M., Knop J., Malmberg A.B., McDermott J.S., Nguyen H.Q., Waldon D., Albrecht B.K., McDonough S.I. (2008). Synthesis and activity of substituted carbamates as potentiators of the alpha4beta2 nicotinic acetylcholine receptor. Bioorg. Med. Chem. Lett..

[26] Wang J., Lindstrom J. (2018). Orthosteric and allosteric potentiation of heteromeric neuronal nicotinic acetylcholine receptors. Br. J. Pharmacol..

[27] Richter L., de Graaf C., Sieghart W., Varagic Z., Morzinger M., de Esch I.J., Ecker G.F., Ernst M. (2012). Diazepam-bound GABAA receptor models identify new benzodiazepine binding-site ligands. Nat. Chem. Biol..

[28] Wang Z.J., Deba F., Mohamed T.S., Chiara D.C., Ramos K., Hamouda A.K. (2017). Unraveling amino acid residues critical for allosteric potentiation of (alpha4)3(beta2)2-type nicotinic acetylcholine receptor responses. J. Biol. Chem..

[29] Mazzaferro S., Bermudez I., Sine S.M. (2019). Potentiation of a neuronal nicotinic receptor via pseudo-agonist site. Cell. Mol. Life Sci..

[30] Indurthi D.C., Lewis T.M., Ahring P.K., Balle T., Chebib M., Absalom N.L. (2016). Ligand binding at the 4-4 agonist-binding site of the 42 nAChR triggers receptor activation through a pre-activated conformational state. PLoS One.

[31] Steinbach J.H., Akk G. (2019). Applying the Monod-Wyman-Changeux allosteric activation model to pseudo-steady-state responses from GABAA receptors. Mol. Pharmacol..

[32] Monod J., Wyman J., Changeux J.P. (1965). On the nature of allosteric transitions: A plausible model. J. Mol. Biol..

[33] Forman S.A. (2012). Monod-Wyman-Changeux allosteric mechanisms of action and the pharmacology of etomidate. Curr. Opin. Anaesthesiol..

[34] Mazzaferro S., Gasparri F., New K., Alcaino C., Faundez M., Iturriaga Vasquez P., Vijayan R., Biggin P.C., Bermudez I. (2014). Non-equivalent ligand selectivity of agonist sites in (alpha4beta2)2alpha4 nicotinic acetylcholine receptors: A key determinant of agonist efficacy. J. Biol. Chem..

[35] Grupe M., Jensen A.A., Ahring P.K., Christensen J.K., Grunnet M. (2013). Unravelling the mechanism of action of NS9283, a positive allosteric modulator of (alpha4)3(beta2)2 nicotinic ACh receptors. Br. J. Pharmacol..

[36] Eaton M.M., Germann A.L., Arora R., Cao L.Q., Gao X., Shin D.J., Wu A., Chiara D.C., Cohen J.B., Steinbach J.H., Evers A.S., Akk G. (2016). Multiple non-equivalent interfaces mediate direct activation of GABAA receptors by propofol. Curr. Neuropharmacol..

[37] Sine S.M., Steinbach J.H. (1984). Agonists block currents through acetylcholine receptor channels. Biophys. J..

[38] Connolly J., Boulter J., Heinemann S.F. (1992). Alpha 4-2 beta 2 and other nicotinic acetylcholine receptor subtypes as targets of psychoactive and addictive drugs. Br. J. Pharmacol..

[39] Giniatullin R.A., Sokolova E.M., Di Angelantonio S., Skorinkin A., Talantova M.V., Nistri A. (2000). Rapid relief of block by mecamylamine of neuronal nicotinic acetylcholine receptors of rat chromaffin cells *in vitro*: An electrophysiological and modeling study. Mol. Pharmacol..

[40] Peters L., Wright A.D., Kehraus S., Gundisch D., Tilotta M.C., Konig G.M. (2004). Prenylated indole alkaloids from Flustra foliacea with subtype specific binding on NAChRs. Planta Med..

[41] Sala F., Mulet J., Reddy K.P., Bernal J.A., Wikman P., Valor L.M., Peters L., Konig G.M., Criado M., Sala S. (2005). Potentiation of human alpha4beta2 neuronal nicotinic receptors by a Flustra foliacea metabolite. Neurosci. Lett..

[42] Li P., Steinbach J.H. (2010). The neuronal nicotinic alpha4beta2 receptor has a high maximal probability of being open. Br. J. Pharmacol..

[43] Wang J., Kuryatov A., Sriram A., Jin Z., Kamenecka T.M., Kenny P.J., Lindstrom J. (2015). An accessory agonist binding site promotes activation of alpha4beta2∗ nicotinic acetylcholine receptors. J. Biol. Chem..

[44] Weltzin M.M., Schulte M.K. (2015). Desformylflustrabromine modulates alpha4beta2 neuronal nicotinic acetylcholine receptor high- and low-sensitivity isoforms at allosteric clefts containing the beta2 subunit. J. Pharmacol. Exp. Ther..

[45] Deba F., Ali H.I., Tairu A., Ramos K., Ali J., Hamouda A.K. (2018). LY2087101 and dFBr share transmembrane binding sites in the (α4)3(β2)2 nicotinic acetylcholine receptor. Sci. Rep..

[46] Walsh R.M., Roh S.H., Gharpure A., Morales-Perez C.L., Teng J., Hibbs R.E. (2018). Structural principles of distinct assemblies of the human alpha4beta2 nicotinic receptor. Nature.

